# Exploring the Involvement of NLRP3 and IL-1*β* in Osteoarthritis of the Hand: Results from a Pilot Study

**DOI:** 10.1155/2019/2363460

**Published:** 2019-03-10

**Authors:** Antonella Fioravanti, Sara Tenti, Megan McAllister, Melody Chemaly, Amanda Eakin, Joseph McLaughlin, Anthony J. Bjourson, Elena Frati, Victoria McGilligan, Sara Cheleschi, David S. Gibson

**Affiliations:** ^1^Rheumatology Unit, Department of Surgery, Medicine and Neurosciences, Le Scotte Hospital, Siena, Italy; ^2^Northern Ireland Centre for Stratified Medicine, University of Ulster, Biomedical Sciences, Research Institute, Londonderry, UK

## Abstract

Hand osteoarthritis (HOA) includes different subsets; a particular and uncommon form is erosive HOA (EHOA). Interleukin- (IL-) 1*β* plays a crucial role in the pathogenesis of osteoarthritis (OA); it is synthesized as an inactive precursor which requires the intervention of a cytosolic multiprotein complex, named inflammasome, for its activation. The aim of this study was to investigate the involvement of IL-1*β* and the NOD-like receptor pyrin domain containing 3 (NLRP3) inflammasome in patients with EHOA and nonerosive HOA (NEHOA) compared to healthy controls. In particular, we evaluated the gene expression of IL-1*β* and NLRP3, the serum levels of IL-1*β*, IL-6, IL-17, and tumor necrosis factor- (TNF-) *α*, and the protein levels of IL-1*β* and NLRP3. We also assessed the relationships between IL-1*β* and NLRP3 and clinical, laboratory, and radiological findings. Fifty-four patients with HOA (25 EHOA and 29 NEHOA) and 20 healthy subjects were included in the study. Peripheral blood mononuclear cell (PBMC) gene and protein expressions of IL-1*β* and NLRP3 were quantified by quantitative real-time PCR and western blot. IL-1*β*, IL-6, IL-17, and TNF-*α* serum levels were determined by ELISA. IL-1*β* gene expression was significantly reduced (*p* = 0.0208) in EHOA compared to healthy controls. NLRP3 protein levels were significantly increased in the NEHOA group versus the control (*p* = 0.0063) and EHOA groups (*p* = 0.0038). IL-1*β* serum levels were not significantly different across the groups; IL-6, IL-17, and TNF-*α* were not detectable in any sample. IL-1*β* concentrations were negatively correlated with the Kellgren-Lawrence score in the whole population (*r* = −0.446; *p* = 0.0008) and in NEHOA (*r* = −0.608; *p* = 0.004), while IL-1*β* gene expression was positively correlated with the number of joint swellings in the EHOA group (*r* = 0.512; *p* = 0.011). Taken together, our results, showing poorly detectable IL-1*β* concentrations and minimal inflammasome activity in the PBMCs of HOA patients, suggest a low grade of systemic inflammation in HOA. This evidence does not preclude a possible involvement of these factors at the local level.

## 1. Introduction

Osteoarthritis of the hand (HOA) is a common form of osteoarthritis (OA), affecting a large percentage of the population over 50 years [1]. HOA is generally considered a heterogeneous group of diseases including different subsets [2]. A particular and uncommon subset of HOA is the so called erosive osteoarthritis of the hand (EHOA) characterized by an abrupt onset, inflammatory signs, and importantly, more disability than nonerosive hand OA (NEHOA) [3]. EHOA mainly affects the distal and proximal interphalangeal (IP) joints with prominent destructive damage, consisting of subchondral erosions and bone ankylosis [4, 5]. The diagnosis of EHOA is commonly based on characteristic radiographical changes including typical central erosions, collapse of the subchondral bone, and the “gull-wing” and/or “saw-tooth” deformity [4]. Laboratory findings, including rheumatoid factor, anticyclic citrullinated peptide antibodies are usually negative, while contrasting data have been reported about erythrocyte sedimentation rate (ESR) and high sensitivity C reactive protein (hsCRP) levels [6–8]. Recent data from various pilot studies showed an increase of biomarkers of joint inflammation such as myeloperoxidase [9–11].

There has been much debate in recent years regarding the role of systemic inflammation in erosive and nonerosive HOA [12, 13]. Different inflammatory cytokines, such as interleukin- (IL-) 1*β*, IL-6, IL-17, and tumor necrosis factor alpha (TNF-*α*), released from various cell types can promote HOA cartilage degradation, synovial inflammation, and disease progression [14–17].

IL-1*β* plays a crucial role in the local pathogenesis of OA leading to the release of cartilage-degrading enzymes, such as metalloproteinases (MMPs) and aggrecanases (ADAMTS-4 and 5), from chondrocytes and inhibiting the production of the extracellular matrix [18, 19]. IL-1*β* is synthesized as an inactive precursor (pro-IL-1*β*) which requires cleavage of its amino-terminal region by caspase 1 to produce the active form. The activation from pro-caspase 1 to caspase 1 requires the intervention of large cytosolic multiprotein complexes, named inflammasomes [20]. NOD-like receptor pyrin domain containing 3 (NLRP3) is the most studied subtype of inflammasome. Inflammasome proteins are mainly expressed in innate immune cells, as monocytes and macrophages, as well as in neutrophils and mesenchymal cells, including osteoblasts and chondrocytes [21]. NLRP3 is activated by danger-associated molecular patterns (DAMPs) and pathogen-associated molecular patterns (PAMPs), such as ATP and crystalline agonists including calcium pyrophosphate dihydrate (CPPD) and monosodium urate (MSU) [22]. Other pathways of activation of pro-IL-1*β* are mediated by some extracellular proteases (trypsin, chymotripsin, cathepsin G, and elastase) or by MMPs, particularly MMP-9 [23].

In the last decade, several studies have highlighted the central role of the NLRP3 inflammasome in the pathogenesis of inflammatory and immune disorders [24]. Conversely, there are few contrasting reports about the involvement of NLRP3 inflammasome in the pathophysiology of OA [25].

The aim of this study was to investigate the possible involvement of IL-1*β* and the NLRP3 inflammasome in patients with EHOA and NEHOA in comparison to healthy controls. In particular, we evaluated the gene expression and the protein levels of IL-1*β* and NLRP3 by quantitative real-time PCR and western blot analysis in the peripheral blood mononuclear cells (PBMCs); in addition, the serum levels of IL-1*β*, IL-6, IL-17, and TNF-*α* by the ELISA assay were also assessed. Furthermore, we investigated the relationships between IL-1*β* and NLRP3 and the clinical, laboratory, and radiological parameters studied in EHOA and NEHOA patients.

## 2. Patients and Methods

### 2.1. Study Population

Fifty-four Caucasian outpatients who fulfilled the American College of Rheumatology criteria for hand osteoarthritis [26] were recruited in the Rheumatology Unit of Siena Hospital from December 2014 to March 2016. All patients underwent radiographic examination of the hands.

Patients were divided into EHOA and NEHOA groups. EHOA was defined by the presence of the classical central erosion in at least two IP joints [4]. We identified 25 EHOA patients and 29 NEHOA. A control group was represented by 20 healthy subjects without hand joint pain and/or tenderness and finger nodes. These subjects did not show clinical signs of OA in any articular joints and were not affected by other autoimmune or inflammatory disorders.

The main exclusion criteria included the presence of inflammatory rheumatic and bowel diseases and personal and/or familial history of psoriasis. Furthermore, patients treated in the last year with immunosuppressants or symptomatic slow-acting drugs for osteoarthritis (SySADOA), such as chondroitin sulphate, glucosamine sulphate, diacerein, avocado/soybean unsaponifiables, and intra-articular hyaluronic acid or nutraceuticals [27], were excluded.

All participants gave written informed consent before inclusion. The study was approved by the Local Ethical Committee (decision no. 30.11.07).

### 2.2. Clinical and Radiographic Assessment

Demographic and clinical data including age, gender, height, weight, BMI, disease duration, concomitant hip and knee OA involvement [28], smoking, and medical history of type II diabetes mellitus, hypertension, cardiovascular (CV) diseases, or autoimmune thyroiditis were collected at the time of enrollment. All patients underwent an anteroposterior X-ray projection of both hands; the radiological assessment was carried out by consensus opinion (joint wise at the same time) by two experienced readers, according to the Kellgren-Lawrence score [29].

Clinical evaluation also included swollen joint count, ESR, serum CRP concentrations, the patient's assessment of spontaneous hand pain on a 0-100 mm visual analogue scale (VAS), and the functional index for hand osteoarthritis (FIHOA) validated in Italian language [30, 31].

### 2.3. Serum Collection and Isolation of PBMCs

Overnight fasting blood samples (6 ml) were obtained from an antecubital vein with the subject in the supine position. The blood was immediately centrifuged, and the serum was stored at -80°C until following analysis. EDTA-treated blood was separated in its fractions by Ficoll (Ficoll-Paque GE Healthcare, UK) density gradient centrifugation to collect the plasma and PBMCs. The total cell lysates were obtained with M-PER™ Mammalian Protein. Extraction Reagent (Thermo Fisher Scientific, Rockford, IL, USA) containing a protease inhibitor cocktail (Sigma-Aldrich S.r.l., Milan, Italy), while the total RNA was extracted from PBMCs using the TRIzol reagent according to the manufacturer's instructions (Thermo Fisher Scientific, Waltham, USA).

### 2.4. ELISA Assay of IL-1*β*, IL-6, IL-17A, and TNF-*α*

IL-1*β* levels were quantified using the Cymax™ Human ultrasensitive IL-1*β* ELISA assay (L.O.D. 1.90 pg/ml; Ab Frontier, Korea).

IL-6 and TNF-*α* serum levels were quantified using the Human IL-6 PicoKine ELISA kit (L.O.D.<0.3 pg/ml) and Human TNF-*α* PicoKine ELISA kit (L.O.D.<1 pg/ml), respectively (Vinci-Biochem srl., Italy).

IL-17 serum levels were assessed by Human IL-17A Platinum Kit ELISA (L.O.D. 1.6 pg/ml; Invitrogen, Thermo Fisher Scientific, Italy).

Reagents and assay procedures were prepared according to the manufacturer's guidelines. The products of the enzymatic reactions were immediately read by a Microplate Spectrophotometer (Tecan Group Ltd., Switzerland) and by a Microplate Reader (BioTek Instruments Inc., USA). A standard curve was created from plate data, and the unknown values were extrapolated in Microsoft Excel.

### 2.5. RNA Extraction and Real-Time PCR

Real-time PCR was used to measure gene expression of NLRP3 and IL-1*β* relative to glyceraldehyde-3-phosphate dehydrogenase (GAPDH).

RNA was extracted from cell pellets which were already stored in TRIzol Reagent (Thermo Fisher, U.S.A.). The concentration of RNA was measured using the NanoDrop 2000 (Thermo Fisher, U.S.A.). cDNA was then reverse transcribed from 80 ng of RNA using the Transcriptor First Strand cDNA Synthesis Kit (Roche Diagnostics, U.K.) on the Prime Thermal Cycler (Techne, U.K.).

Real-time PCR was performed using the LightCycler 480 II and the LightCycler 480 Probe Master Reagents and probes (Roche, Switzerland). PCR grade water, a minus RT control, a probe control (probe, probe master mix control, and water), and a cDNA control (cDNA template, PCR grade water, and probe master mix) were run on each plate. Gene expression was normalized using GAPDH as a reference gene, and relative expression values were determined using the delta delta Ct calculation method, then plotted as ratios of healthy control values.

### 2.6. Western Blot Analysis

Western blot analysis was used to quantify the protein levels of IL-1*β* and NLRP3 relative to *β*-actin.

Protein concentration of PBMC samples was determined using Pierce BCA Protein Assay Kit (Thermo Scientific, USA) to facilitate equal lane loading on western blot. IL-1*β* and NLRP3 proteins were quantified using NuPAGE Electrophoresis System. Protein (17 *μ*g) from each sample was loaded per lane and electrophoresed in 1xNuPAGE (MOPS) SDS Running Buffer on NuPAGE 4-12% BisTris Gels. 500 *μ*l of NuPAGE antioxidant per gel was used in the inner chamber. Following electrophoresis, proteins were transferred onto a PVDF membrane in 1x transfer buffer (20xNuPAGE Transfer Buffer, 20% methanol, dH20, NuPAGE antioxidant). Membranes were blocked in 5% Marvel and PBST for one hour. Running buffer, gels, antioxidant, membranes, and transfer buffer were purchased from Life Technologies, USA. Membranes were also exposed to anti-IL-1*β* goat IgG (R&D, USA) diluted 1 : 200 in PBS overnight at 4°C, washed in PBST, and incubated with rabbit anti-goat IgG-HRP (Santa Cruz Biotechnology) diluted 1 : 2000 in PBS for one hour at room temperature (a human recombinant IL-1*β* positive control protein sample was used to verify the method). Membranes were developed using SuperSignal West Pico Chemiluminescent Substrate (Thermo Scientific, USA) and imaged using UVP ChemiDoc-It2 Imager and VisionWorks Analysis Software version 7.1. *β*-Actin was used as a loading control.

Background subtraction was done, and band densitometry was used to normalize IL-1*β* and NLRP3 to *β*-actin for each sample.

### 2.7. Statistical Analysis

Continuous parameters are expressed as mean and standard deviation (SD); categorical variables are expressed as frequency and percentages.

Comparison for continuous normally distributed clinical, demographic, and biochemical variables between two groups was performed by *t*-test and among three groups by the analysis of variance (ANOVA) test; the nonparametric Mann-Whitney test was used for nonnormal variables. Frequency comparisons between two groups and three groups were analyzed using the *χ*^2^ (chi square) test and contingency table method, respectively.

Correlations were obtained and expressed as Pearson correlation coefficient (*r*), *α* (two-sided) was set at 0.05, and study power = 95%. Statistical analysis was performed with Prism 6.01 (GraphPad Software Inc., California, USA). For all tests, a *p* value <0.05 was considered as statistically significant.

## 3. Results

### 3.1. Patients Characteristics

Demographic and clinical characteristics of the different study groups are summarized in Table 1. Our study population was mainly represented by female patients (78.3%); Kellgren and Lawrence radiological grades were equally distributed between EHOA and NEHOA patients. The three groups were also comparable for sex distribution, BMI, frequency of diabetes, CV diseases, autoimmune thyroiditis, concomitant knee/hip OA, and number of hand joint swellings. However, subject age, frequency of smoking, and hypertension significantly differed among the three groups (*p* = 0.001, *p* = 0.0110, and *p* = 0.0012, respectively). Particularly, the control group was represented by subjects younger than those affected by EHOA and NEHOA (*p* < 0.001) with a lower percentage of concomitant hypertension (*p* < 0.001) and a greater frequency of smokers (*p* = 0.007 and *p* = 0.035, respectively).

Concerning laboratory measurements, no differences were found in ESR values among the three groups. Conversely, for CRP, we observed a significant difference among the three groups (*p* = 0.0125) and between the EHOA and NEHOA groups (*p* = 0.003).

The disease duration, the assessment on 0-100 mm VAS pain, and the algofunctional index, FIHOA, were significantly greater in EHOA versus NEHOA (*p* = 0.0175, *p* = 0.0039, and *p* = 0.0020, respectively).

### 3.2. Circulating Levels, Gene Expression, and Protein Levels of IL-1*β*

**Figure 1:**
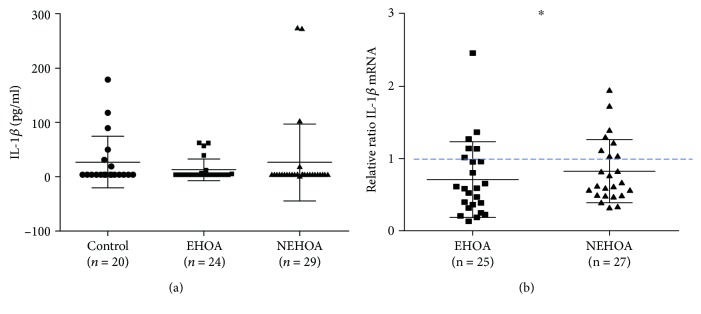
Evaluation of IL-1*β* at the serum level (a) by the ELISA assay and at the gene expression level (b) by real-time PCR, in a control group, patients with erosive hand OA (EHOA), and patients with nonerosive hand OA (NEHOA). The gene expression was normalized to controls. Data are expressed as mean ± standard deviation. ^∗^*p* < 0.05 versus control group.

IL-1*β* measured by the ELISA assay showed no significant differences among the three studied groups (Figure 1(a)).  Figure 1(b) indicates the analysis of IL-1*β* by quantitative real-time PCR. IL-1*β* gene expression was significantly lower (*p* = 0.0208) in EHOA patients in comparison to the control group; no significant differences were observed in comparison to the NEHOA group. Patients with NEHOA showed slightly lower IL-1*β* expression levels than controls, although in a not significant manner. Three samples were not analyzed as the extracted RNA quality was poor, and another 3 samples were not included in the analysis as Ct values were over 40. IL-1*β* protein expression was not detected by western blotting (data not shown).

### 3.3. Gene Expression and Protein Levels of NLRP3

**Figure 2:**
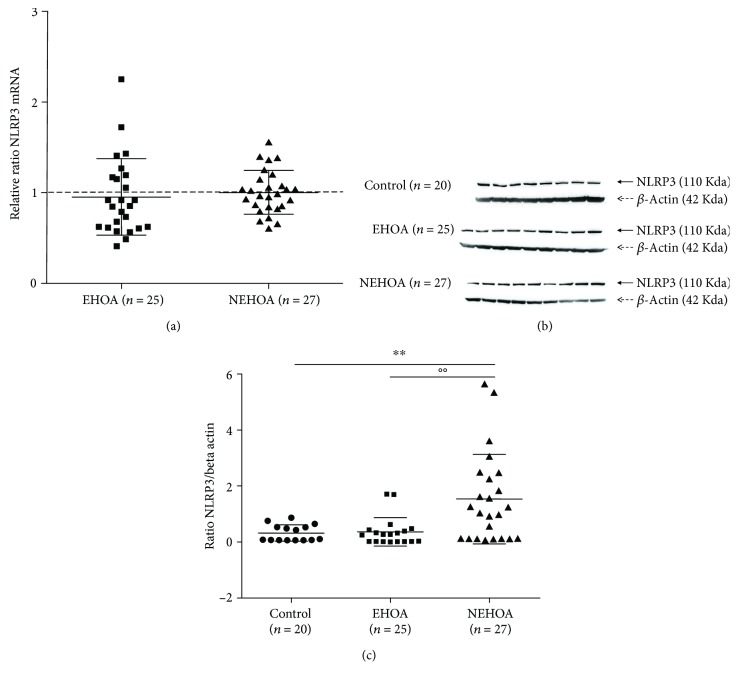
Evaluation of NLRP3 gene expression (a) by real-time PCR and of NLRP3 protein levels (b and c) by western blot analysis in a control group, patients with erosive hand OA (EHOA), and patients with nonerosive hand OA (NEHOA). The gene expression was normalized to controls. Data are expressed as mean ± standard deviation. ^∗∗^*p* < 0.01 versus control group; °°*p* < 0.01 EHOA group versus NEHOA group.

In the PBMCs of the three groups, there was no statistical difference of NLRP3 gene expression (Figure 2(a)). Three samples were not analyzed as the extracted RNA quality was poor.

Figures 2(b) and 2(c) showed the western blot analysis of NLRP3. Some samples were excluded as protein concentrations measured by the BCA assay were not sufficient. The densitometric quantification of the bands showed a significant increase (*p* = 0.0063) of NLRP3 protein levels in the NEHOA group in comparison to controls. In addition, NLRP3 protein levels were significantly higher in the NEHOA group (*p* = 0.0038) than in the EHOA group. No significant differences were observed between EHOA patients and controls.

### 3.4. Serum Levels of IL-6, IL-17A, and TNF-*α*

The ELISA assay carried out on IL-6, IL-17A, and TNF-*α* serum levels resulted in nondetectable levels for all samples analyzed (data not shown).

### 3.5. Correlation Analysis between IL-1*β* and NLRP3 and the Other Parameters Studied

**Figure 3:**
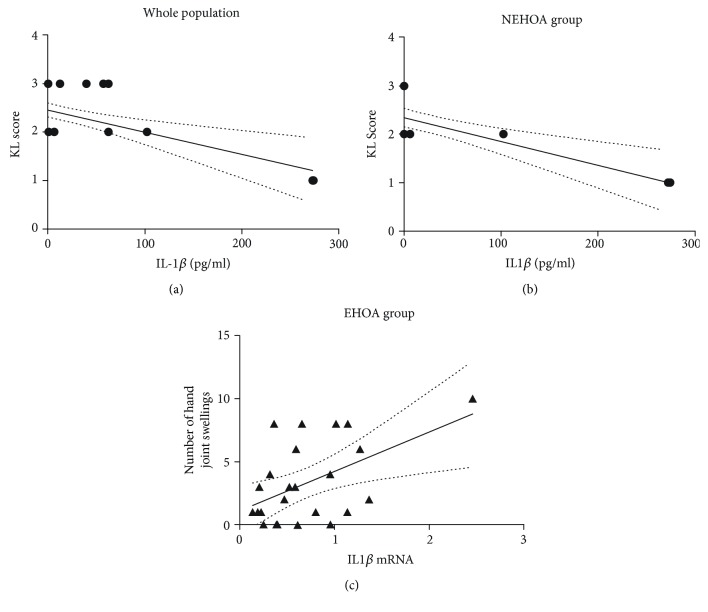
Graphical representation of the found correlations between IL-1*β* serum levels and the Kellgren-Lawrence (K-L) score in the whole population (a; *r* = −0.446, *p* = 0.0008) and in the NEHOA group (b; *r* = −0.608, *p* = 0.004) and IL-1*β* mRNA levels and the number of joint swellings in the EHOA group (c; *r* = 0.512, *p* = 0.011).

Figure 3 shows the existing correlations between serum levels and gene expression of IL-1*β* and the Kellgren-Lawrence score and the number of hand joint swellings, respectively. In particular, IL-1*β* concentrations were negatively correlated in a significant manner with the radiological grade, measured by the Kellgren-Lawrence score in the whole population (*r* = −0.446; *p* = 0.0008) and in the NEHOA group (*r* = −0.608; *p* = 0.004) (Figures 3(a) and 3(b)).

IL-1*β* gene expression significantly and positively correlated with the number of hand joint swellings only in the EHOA group (*r* = 0.512; *p* = 0.011) (Figure 3(c)). No other correlations were observed either between IL-1*β* and the other evaluated parameters or between gene expression and protein levels of NLRP3 and the clinical, laboratory, and radiological findings in the whole population and in each subgroup.

## 4. Discussion

To the best of our knowledge, this is the first study to investigate more deeply the involvement of IL-1*β* and NLRP3 in PBMCs of patients with two clinically important subsets of HOA, erosive and nonerosive forms.

Despite the fact that many *in vitro* studies have well documented the important contribution of IL-1*β* to the OA pathogenesis, the role of this cytokine in the clinical setting has not been fully elucidated. On one hand, IL-1*β* has been shown to affect cartilage homeostasis and to drive the synthesis of proteolytic enzymes, such as MMPs, ADAMTs, and the production of other cytokines such as IL-8, IL-6, and IL-17 [32, 33]. Conversely, data from different OA animal models reported discordant results; mice deficient in IL-1 developed more severe cartilage lesions than wild-type (WT) mice [34], and intraperitoneal injections of IL-1 antagonists did not improve the OA injuries in a meniscectomy-induced murine model of OA [35]. Furthermore, clinical evidence demonstrated very low levels of IL-1*β* in the synovial membrane and fluid of patients affected by early- and end-stage OA [36].

A potential role for IL-1*β* in the pathogenesis of HOA, in particular in the severe subset of EHOA, was suggested by some authors [37, 38]. Stern et al. [37] reported the association between a single nucleotide polymorphism (SNP) identified on the gene-encoding IL-1*β* and the development of EHOA.

Surprisingly, in our study, IL-1*β* gene expression was marginally lower in erosive patients than nonerosive patients although this wasn't statistically significant. There was, however, a statistical difference between the control group and the erosive group, with higher IL-1*β* expression seen in the controls. Protein levels of IL-1*β* resulted not detectable in all samples, and its serum levels were around the limits of detection without any difference among the three groups.

Currently, it is difficult to explain the discrepancy between the results obtained at IL-1*β* gene expression and serum levels. We can postulate that this difference may be due to the increase in the percentage of IL-1*β* bound to its receptors. Intriguingly, a higher density of IL-1 receptors has been reported in osteoarthritic chondrocytes compared to healthy controls [39]; therefore, it would be interesting to compare receptor density between erosive and nonerosive patients to verify our hypothesis. Furthermore, it was demonstrated that the release of IL-1 occurred in the early phases of OA, and this could represent another explanation for the low IL-1*β* serum levels described in this paper, considering that mean disease duration of our study population was higher than 8 years [38]. Alternatively, we can hypothesize a compensatory mechanism in place to dampen the inflammatory response in the EHOA and NEHOA patients.

Considering together, the results derived by IL-1*β* serum levels and gene and protein expressions, an independent mechanism from IL-1 pathways in cartilage degradation and inflammation could be also supposed.

Recent evidence corroborates our results showing that in meniscectomized knock out mice for IL-1*β* and inflammosome complex, the severity of cartilage lesions was similar or worse than in meniscectomized WT mice [34, 40]. Besides the pivotal role of IL-1*β* in OA, it has also been targeted with the IL-1 receptor antagonist (IL-1Ra) in randomized clinical trial (RCT) patients with knee OA. Both intra-articular anakinra, a recombinant form of IL-1Ra, and subcutaneous use of AMG108, a monoclonal antibody against IL-1R1, failed to demonstrate significant clinical efficacy [41, 42]. Concerning HOA, the data are limited to two different reports of 3 cases of severe EHOA successfully treated with subcutaneous injections of anakinra [43, 44].

In the current study, we analyzed the possible involvement of NLRP3 in erosive and nonerosive HOA.

NLRP3 is an intracellular complex that mediates the cellular response to exogenous and endogenous pathogens, regulating the release of proinflammatory cytokines, mainly IL-1*β*. Thus, the inflammasome plays a paramount role not only in the process of host protection against infective agents, but also in the rheumatic inflammatory diseases [45]. However, the role of NLRP3 in OA appears highly controversial. Some studies have reported an overexpression of NLRP3 protein in synovial tissue from OA joints compared to controls and a positive correlation with nicotinamide adenine dinucleotide phosphate oxidase- (Nox-) 2, a prooxidant enzyme involved in oxidative stress [46, 47]. Other studies suggested that OA cartilage degradation occurs independently of NLRP3 inflammasome activity [40, 48]. In particular, Bougault et al. [48] found that the IL-1*β* release from cartilage explants of 18 knee OA patients who underwent total joint replacement was poorly detectable and lower than synovial samples. The authors also reported the protein and gene expressions of NLRP3, ASC, and caspase-1 in human OA chondrocytes, although they seemed to acquire a prodegradative phenotype without any contribution by NLRP3 and IL-1*β*. Indeed, the protein and gene expressions of MMPs in knock out (NLRP3^−/−^) and WT mice did not differ, and the caspase-1 inhibition leads to nonsignificant modifications. Similar results were also obtained after biochemical load stimulation of mouse cartilage explants.

Our results did not show any significant difference in NLRP3 gene expression between the three subgroups. Conversely, NLRP3 protein expression was significantly higher in the NEHOA group compared to EHOA and controls. The different pattern showed by gene expression and protein levels of NLRP3 could be due to posttranslational modifications or mRNA instability which could affect protein expression [49, 50]. Using reverse transcription-PCR, NLRP3, ASC, caspase-1, and pro-IL-1*β*, messenger RNA was detected in OA synovial tissue in either all cases or in the majority of cases of a previous study [51]. Protein expression of NLRP3 was confirmed by western blot, which is in line with the current results showing NLRP3 expression in PBMC samples of those with OA [51].

In our study, we showed low NLRP3 protein expression in EHOA patients compared to NEHOA and controls, even if EHOA is characterized by clinical inflammatory features and radiographic erosions, as main hallmarks. Presently, it is very difficult to explain these results.

One possible hypothesis is the higher, although not statistically significant, frequency of certain comorbidities, such as type II diabetes, CV diseases, and autoimmune thyroiditis in the nonerosive group compared to the erosive group. There is evidence suggesting these conditions enhance the NLRP3 inflammasome expression [52–54]. We have also to take into consideration that the control group included a higher percentage of smokers compared with both EHOA and NEHOA patients. It was demonstrated that nicotine exposure could induce NLRP3 inflammasome activation in endothelial cells, mainly as a consequence of generation of reactive oxygen species (ROS) and an increased IL-1*β* gene expression in lung tissue extracts [55, 56]; moreover, an increase of IL-1*β* concentrations was observed in smokers' sera [57]. On the other side, recent studies showed that cigarette smoke extract decreased NLRP3 protein levels, through an increase of ubiquitin-mediated proteasomal processing, and subsequently reduced the release of IL-1*β* in human THP1 monocytes [58]. Thus, at the moment, it is difficult to clarify the exact effect of smoking on NLRP3 and IL-1*β* levels. It is also important to highlight that our study analyzed the protein and gene expressions of IL-1*β* and NLRP3 in PBMCs and not in cartilage cells. This method can have some limitations in a disease characterized by a prominent local inflammation, such as EHOA, because PBMCs reflect the inflammatory state at a systemic level [12] Thus, it is very difficult to obtain cartilage and synovial samples from IF joints, and although monocytes, macrophages, and dendritic cells are the main NLRP3-expressing cells, human neutrophils have also shown to have functional NLRP3 inflammasome [59]. Furthermore, PBMCs have been considered as a common source of genomic material for microarray studies, mainly due to the facility and relative noninvasiveness of acquisition [60].

To understand if pathways other than those mediated by NLRP3 inflammasome may be involved in the pathogenesis of the two considered subsets of HOA, we measured the serum levels of other known proinflammatory cytokines, including IL-6, IL-17, and TNF-*α*. These cytokines were not present at detectable levels (stating the lowest level of detection of the assays).

Our IL-6 results concur with previous studies, in patients with HOA where circulating IL-6 levels were not statistically different among NEHOA, EHOA, and healthy subjects [61, 62]. Conversely, others have demonstrated high synovial and serum levels of IL-6 in subjects with knee OA in comparison to the healthy controls [14, 63]. This inconsistency could be explained by the evidence that the infrapatellar fat pad is a source of cytokines, as IL-6, while the small joints of the hand are characterized by a minor amount of adipose tissue [64].

A recent hypothesis defines IL-17 as a peculiar inflammatory OA phenotype; in this regard, we analyzed the serum levels of this cytokine in our HOA study groups [65, 66]. Unexpectedly, we detected very low concentrations of IL-17 in our HOA population, without any difference among the examined groups. Others have described increased IL-17 serum levels in patients with knee OA compared to healthy controls; however, no data have been previously published in HOA [67–69].

Concerning serum levels of TNF-*α*, we observed no difference among the three analyzed groups. To the best of our knowledge, there are no previous papers exploring the circulating TNF-*α* levels in patients with EHOA, and data derived from knee OA are controversial [63, 70]. After all, the clinical studies aimed at assessing the efficacy of TNF-*α* inhibitors (TNF-*α*i) in subjects with HOA are too heterogeneous to draw any clear conclusions. Indeed, three RCTs enrolling HOA patients treated with adalimumab failed to show the superiority of the TNF-*α*i over placebo [71–73]. On the other hand, a recent RCT reported encouraging results on etanercept therapy in an EHOA population [74]. Also, previous open-label trials provided controversial outcomes; Magnano et al. [75] observed no significant improvements, after treatment with adalimumab in 12 patients with EHOA, while a single-blind study on 10 EHOA patients suggested a clinical benefit of intra-articular therapy with infliximab [76].

We observed a negative correlation between IL-1*β* serum levels and the radiographic Kellgren-Lawrence score in the whole population and in the NEHOA group, but surprisingly, it was not confirmed in EHOA patients. The lack of a correlation in the EHOA group may be due to the missing data regarding other radiological grades, as the Verbruggen and Kallman scores, considered superior in measuring the erosive lesions to the Kellgren-Lawrence [38]. This hypothesis can be supported by the results reported by Bondeson et al. [32]; in fact, the authors demonstrated a negative significant correlation between IL-1 serum levels and Verbruggen and Kallman scores in EHOA patients but not with Kellgren-Lawrence.

Furthermore, we detected a positive and significant correlation between IL-1*β* gene expression and the number of hand joint swellings in the EHOA group. This result may reflect the disease activity, as demonstrated by the significantly higher CRP levels in our erosive patients than NEHOA.

However, we are aware that our study presents several limitations. Firstly, the number of patients is small, and the three groups were not homogeneous for all the demographical and clinical measures; in particular, the controls were younger than both the other study groups and maybe for this reason presented a minor frequency of hypertension. They were also smokers in a greater percentage than HOA patients, and this could affect our IL-1*β* and NLRP3 results. Secondly, the longer disease duration in EHOA patients compared to the NEHOA group could have influenced the gene and protein expressions of NLRP3 and IL-1*β*. Furthermore, the use of the radiographic scores of Verbruggen and Kallman should allow to obtain a more appropriate assessment of erosive lesions in EHOA.

Finally, our results may have been limited by the missing analysis of inflammasome subtypes other than NLRP3 and its multiple polymorphisms and measure of IL-18 another major cytokine released via the NLRP3 pathway.

All these limitations, particularly the lack of homogeneity among the three groups, induce us to consider our results as preliminary and to advocate further studies in this field.

## 5. Conclusions

This study showed low serum, protein, and gene expression levels of IL-1*β* in EHOA and NEHOA patients; we have also observed low NLRP3 protein expression in EHOA patients compared to NEHOA and controls, suggesting the hypothesis of a nonpivotal role for NLRP3 in the pathogenesis of EHOA.

Furthermore, IL-6, IL-17, and TNF-*α* serum levels resulted not detectable in all samples analyzed.

Finally, we found a negative correlation between IL-1*β* serum and the radiological grade, measured by the Kellgren-Lawrence score in the whole population and in the NEHOA group and a positive correlation between IL-1*β* gene expression and the number of hand joint swellings in the EHOA group.

Taken together, our results, showing poorly detectable concentrations of IL-1*β* and minimal inflammasome activity in the PBMCs of patients with HOA, suggest a low grade of systemic inflammation in HOA. This evidence does not preclude a possible involvement of these factors at the local level.

## Figures and Tables

**Table 1:** Demographic and clinical characteristics of the study populations.

	EHOA (*N* = 25)	NEHOA (*N* = 29)	Control (*N* = 20)	*p* value
Age (years)	70.16 ± 8.23	65.44 ± 10.26	52.15 ± 7.49	0.001^a^°
Sex, no. of male/female	3/22	7/22	6/14	0.2606^b^
BMI (kg/m^2^)	25.89 ± 3.62	24.15 ± 3.04	24.46 ± 4.76	0.0603^a^
*Radiographic score (K-L grade) no. (%)*				
I	0 (0)	2 (7)	NA	0.1876^c^
II	11 (44)	18 (62)	NA	0.1910^c^
III	14 (56)	9 (31)	NA	0.0663^c^
Smoker no. (%)	1 (4)	3 (10)	7 (35)	0.0110^b#^
Diabetes no. (%)	4 (16)	6 (21)	1 (5)	0.310^b^
CV disease no. (%)	2 (8)	3 (10)	0 (0)	0.3496^b^
Hypertension no. (%)	12 (48)	12 (41)	0 (0)	0.0012^b##^
Autoimmune thyroiditis no. (%)	4 (16)	7 (28)	0 (0)	0.0643^b^
HOA+knee/hip OA no. (%)	6 (24)	4 (13)	NA	0.5675^c^
Disease duration (months)	152.32 ± 78.39	96.20 ± 88.13	NA	0.0175^d^
No. of hand joint swellings	3.52 ± 3.25	1.75 ± 4.27	NA	0.0966^d^
ESR (mm/h)	21.8 ± 14.61	17.93 ± 10.55	18.05 ± 12.01	0.2651^a^
CRP (mg/dl)	0.39 ± 0.32	0.16 ± 0.20	0.22 ± 0.33	0.0125^a^°°
VAS pain (0-100 mm)	38.8 ± 26.20	19.77 ± 19.98	NA	0.0039^e^
FIHOA (0-30)	10.52 ± 5.78	5.65 ± 5.23	NA	0.0020^e^

EHOA: erosive osteoarthritis of the hand; NEHOA: nonerosive osteoarthritis of the hand; NA: not applicable; BMI: body mass index; K-L grade: Kellgren-Lawrence grade; CV: cardiovascular; HOA: osteoarthritis of the hand; ESR: erythrocyte sedimentation rate; CRP: C reactive protein; VAS: visual analogue scale; FIHOA: functional index for hand osteoarthritis. *p* values <0.05 were considered significant. ^a^ANOVA test for multiple comparison; ^b^3 × 2 contingency table method; ^c^chi square test; ^d^Mann-Whitney test; ^e^unpaired *t*-test. °EHOA group vs. control group: *p* < 0.001; EHOA group vs. NEHOA group: *p* = 0.071; NEHOA group vs. control group: *p* < 0.001 (*p* value was estimated by *t*-test). ^#^EHOA group vs. control group: *p* = 0.007; EHOA group vs. NEHOA group: *p* = 0.374; NEHOA group vs. control group: *p* = 0.035 (*p* value was estimated by the chi square test). ^##^EHOA group vs. control group: *p* = 0.0003; EHOA group vs. NEHOA group: *p* = 0.625; NEHOA group vs. control group: *p* = 0.0009 (*p* value was estimated by the chi square test). °°EHOA group vs. control group: *p* = 0.135; EHOA group vs. NEHOA group: *p* = 0.003; NEHOA group vs. control group: *p* = 0.378 (*p* value was estimated by *t*-test).

## Data Availability

The data used to support the findings of this study are available from the corresponding author upon request.
